# Liver!

**Published:** 1870-04

**Authors:** 


### LIVER!

BY DR. NED.

DEAR DOCTOR:—I have concluded that, at some day not far distant, I would write a book which, I am confident, will be generally read and appreciated. It will be a book filled with pain and pleasure, fear and bravery, health and disease, life and death. It will point out all the causes of success and failure in life, as well as the reasons for crime and debauchery. It will designate the ways of wisdom and folly, will give the solution of all medical questions and will show beyond doubt, the source from whence arises all the disease incident to life. I know that you will at once say that it will be a curious production; but I can write it, I am sure, and I know what the title will be, of what subject I will speak, and from what stand-point I shall make observation. My book will be called LIVER! The one subject treated, all the way through, will be *Liver!* You see, I am anxious to be in fashion; we have educated and scientific physicians all over the world, and all of them are as busy as they *can* be working at the liver. I feel obliged to stick to my professional brethren. We all keep in the same tracks you know, and travel exactly the same road. Our societies and organizations are all based upon the one idea, viz; "We know it all." We build the walls around our members and teach them that we are infallible, and that they must keep strictly within the circumscribed limits, giving them to understand that when they have grown strong and vigorous in ignorance and error, and old in folly and conceit, that they shall be advanced to the watch towers upon the walls, there to sit, like ferocious curs, to growl and whine and bark at everything not bearing the unmistakable sanctity and odor of antiquated dogmas. The point from which I shall make utterance will be *liver*; and, in order to keep the matter before us, I shall suggest, for breakfast—liver, fried; for dinner—liver, stewed; for tea—liver, cold; and for supper—minced liver. You may smile a little, at my liver subject, and so may your readers, but, I tell you seriously, that the liver is a very important thing in the animal economy, for every body has one, or rather, I should say, that every liver has got somebody attached to it!

Anatomists have made a great mistake about this liver, for, they tell us that it is located in the right hypocondriac region; now, that is all nonsense, for I know people who have the liver in their bowels; some in the back; and others again, in the head. In some instances I have known it to get down to the feet, and ache and pain like fury. Then, too, we are told that the liver weighs three or four pounds, and that its office is to secrete bile, etc. These ideas are extremely simple. How in the world could a few pounds be all over a person, who weighed as many hundred? Without doubt the office of the liver is to stir up strife and contention in the system; besides, it is a grand scape-goat for every quack and pretender as well as for every puzzled professor, who looks wise as he attempts to explain the causes of disease and effect the removal of the same, regardless of the symptoms manifested. And here let me remark, that doctors have misled the people, who often know as much as they do themselves, about the *causes* of morbid actions. For, the whole thing is almost entirely speculative, and neither know anything more about the causes of disease than misers do of practical piety, and, as you go *up* in the scale of intellectual merit and wisdom, you hear but little of the causes, but when you come *down*, the twaddle increases at each step, until you reach the very bottom, when you find perfect familiarity with *causes.*

It is truly wonderful what a host of philosophers you will get amongst at the bottom of the ladder. Our best writers and most critical observers tell us that we know but little of the mysterious origin of physical disturbances, and they mourn and lament over their limited abilities, but, when we descend to the professional pigmies, they know *all* about it, and are chanting liver! liver! liver! This gland is, of all things, the most important to the patient, as well as to the doctor; for, neither could get along without a liver to complain about and scold at. Look at it for a moment—a patient presents himself, who complains of pain in his stomach, the doctor *knows* that the cause of it is the liver. Another has pain in the back,—it is the liver. The head aches,—the fault is in the liver. He is insane,—straightway the ugly liver has gone into his head. He has a bad taste in his mouth,—the naughty liver has crept there. The bowels are constipated,—the unruly liver has been doing a bad business. He has diarrhœa,—the liver is only to blame. His complexion is sallow,—it is the liver that makes it so. He is pale as lard,—the liver is in his face. He vomits,—the liver is wrong. He does not vomit,—it is his liver. His feet are cold,—that all arises from the liver; or they burn like fire,—it is his liver. He is cross and morose,—it is his liver. He fights or runs away,—his liver sets him on, or the liver's legs carry him off. His appetite is inordinate, or he cannot eat anything, he sleeps all the time or cannot sleep at all,—why, his great, big liver must be treated! If we made up our mind as to the liver from the complaints of our patients or from the doings of doctors, we should be driven to the conclusion that about nine-tenths of everybody was liver, and the balance nonsense. The people—the masses, tell the truth; and, they exactly reflect the ideas of the profession; they speak just as they are taught, and, accordingly, think that about all diseases arise from the liver, and that everything and everybody is bilious. Just this one error has led to the abuse of one of the most valuable agents in the whole materia medica—mercury. It is supposed to have considerable power in removing bile from the liver, and acting upon the one idea, that the liver can only be blamed in all cases, mercury has become the universal panacea for all disease. No matter if the head, the eyes, the ears, the mouth, throat, stomach, liver, kidneys, the nerves, muscles or tendons, the heart, arteries or veins are affected, mercury becomes the remedy. If the patient is hot or cold, sleepy or wakeful; if he vomits or purges, no matter *what* the disease, he must have his requisite amount of the precious metal. And the guardian of the public health stands by the bed of suffering, with a face as long as a horse's, and with a look as wise as though Solomon were his uncle, and orders his miraculous remedy to go first here, and then there, to effect this organ, and then that, and finally to cure *everything* that comes in its way; and, if needs be, to perform the most wonderful feat, of being at any time suddenly changed from a purgative to a gentle laxative, from a laxative to an alterative, from an alterative to a diuretic, from a diuretic to an anthelmintic, and so on, as the demand may arise. The truth is, we want above all things, honesty, fairness, liberality and humanity in the profession, and then we will be ready to study, investigate and explore, or be willing to bow down before the Omnipotence of Nature's God and own our inability to understand him.

---

Three advanced females—Mrs. Walker, Mrs. Thomas and Mrs. Cumberland—are attending medical lectures at Indianapolis.

